# Fungal septic arthritis in an immunocompetent girl

**DOI:** 10.1186/1546-0096-10-S1-A39

**Published:** 2012-07-13

**Authors:** Kishore C Warrier, Mark Friswell

**Affiliations:** 1 Royal Victoria Infirmary, Newcastle upon Tyne, Tyne and Wear, UK

## Purpose

To look into a case of primary fungal septic arthritis in an immunocompetent girl initially diagnosed and treated as juvenile idiopathic arthritis.

## Methods

A retrospective study of the clinical notes, reports of laboratory investigations and radiological images.

## Results

A 14-year-old girl was referred with pain and swelling in her right knee. This was first noticed 3 months earlier after she banged the knee while swimming. There was no involvement of other joints or systemic symptoms. There was no history of significant illness in the past. Examination confirmed active arthritis of the right knee. Other joints were normal with no systemic signs. With a provisional diagnosis of oligoarticular Juvenile Idiopathic Arthritis, her right knee was injected with Triamcinolone hexactonide. The knee did not respond to the steroid injection and she had significant bruising around the injection site. She underwent further blood tests including tests for Lyme disease and Tuberculosis which were negative, after which her right knee was injected again with steroid after aspirating synovial fluid. The MRI scan confirmed knee effusion with some erosive areas. The synovial aspirate was negative for tuberculosis but grew a fungus of doubtful significance on prolonged culture, later identified as Phaeoacremonium spp. It was revealed that during horse riding while on holiday in Dominican Republic a year earlier, she had scraped her right knee against a tree sustaining an abrasion, but there was no swelling of the knee till the swimming pool accident. The arthritis progressively got worse along with systemic symptoms like high temperature. Her CRP was 39mg/L and ESR 48mm/hr. Her joint was aspirated again, which drained pus. She underwent arthroscopic lavage and debridement. The lateral tibial and femoral condyles were autodigested and she underwent partial synovectomy. She was commenced on intravenous antibiotics with Voriconazole because of the growth of the fungus in April. The aspirates grew Phaeoacremonium again and the bacterial cultures were negative confirming the diagnosis of fungal septic arthritis. She was tested extensively for immunodeficiencies, the results of which were normal. She was continued on systemic antifungal treatment for over one year and the knee improved steadily.

## Conclusion

Herewith reporting the first case to our knowledge of septic arthritis with Phaeoacremonium spp in an immunocompetent child. Phaeoacremonium spp are unusual causes of human disease causing subcutaneous infection to fungaemia, following traumatic implantation of the fungus1. The first case of septic arthritis reported 2 specifically mentions periarticular injury in a subtropical region as a risk factor, which in this case could have been the horse riding injury. Most of the reported cases involve immunocompromised patients 3,4,5 or transplant recipients; which was not the case here. We should consider fungal arthritis in patients presenting with insidious onset monoarthritis especially following trauma.

## Disclosure

Kishore C. Warrier: None; Mark Friswell: None.

